# Global Trends and Future Prospects of Child Nutrition: A Bibliometric Analysis of Highly Cited Papers

**DOI:** 10.3389/fped.2021.633525

**Published:** 2021-09-09

**Authors:** Yunhua Wang, Qiaorong Liu, Yongcong Chen, Yaling Qian, Bei Pan, Long Ge, Qi Wang, Guowu Ding, Jiancheng Wang

**Affiliations:** ^1^School of Management, Lanzhou University, Lanzhou, China; ^2^Gansu Provincial Hospital, Lanzhou, China; ^3^Department of Social Medicine and Health Management, School of Public Health, Lanzhou University, Lanzhou, China; ^4^Evidence Based Social Science Research Center, School of Public Health, Lanzhou University, Lanzhou, China

**Keywords:** child nutrition, bibliometric analysis, highly cited papers, intestinal microflora, obesity

## Abstract

Child nutrition has always been a global concern. This study performed visual analysis of 1,398 child nutrition highly cited papers (HCPs) from 2009 to 2019. The purpose of the study was to evaluate and present the performances of authors, journals, countries, institutions, top cited papers; to explore the hot topics, prospects, and to propose the future research directions on child nutrition. We used bibliometric methods to conduct in-depth statistical analysis of HCPs on child nutrition, showing research progress, trends and hot spots. We included HCPs on child nutrition from the Science Citation Index-Expanded (SCI-E) database February 7, 2020. Two tools, CiteSpace and VOSviewer, were used to conduct the bibliometric analyses. The results showed that, since 2011, the number of HCPs on child nutrition has increased rapidly. The top three contributors in this field were the USA, the UK and Canada. However, the contribution of developing countries was very limited. Intestinal microflora, food allergy, overweight and obesity were the three major research hotspots in this field. Results of this study provide valuable references for ongoing child nutrition related research, which may be interesting and noteworthy to the researchers involved.

## Introduction

Child nutrition has always been a global concern. The United Nations International Children's Emergency Fund (UNICEF) released a report in 2019 about children, food and nutrition, entitled “The State of the World's Children in 2019” (1). The report mentioned that one-third of the world's children under the age of five still cannot get the nutrition they need to grow up currently. At the same time, the burden of malnutrition has become increasingly prominent (2–4). Among the global children under five, there are still 149 million stunted, and nearly 50 million children are in a state of wasting. Three hundred and forty million children face vitamin and mineral deficiencies, which is also known as “hidden hunger” (5). The problem of overweight is developing rapidly (6). Lack of necessary nutrients which may weaken the immune system, cause visual and hearing impairments and may also cause obesity. Studies have shown that the average lifetime income loss per child with growth retardation was $ 1,400, and in developed countries, it was as high as $ 30,000 (7). Overweight and obesity-related diseases, including heart disease, cancer, diabetes, and chronic respiratory diseases, was projected to cost more than $7 trillion in low and middle-income countries between 2011 and 2025 (7–12).

Carrying out scientific research on child nutrition can guide children to take in nutrition reasonably and promote children's healthy growth and development. Black's et al. paper “Global, regional, and national causes of child mortality in 2008: a systematic analysis” conducted a systematic analysis of global causes of child mortality in 2008, and found that nutrition was crucial to guide global efforts to improve child survival (13). Pries's et al. paper “Snack food and beverage consumption and young child nutrition in low–and middle–income countries: A systematic review” believed that although snacks could provide important nutrients for young children during the complementary feeding period, consumption of energy-dense, nutrient-poor snack foods and sugar-sweetened beverages (SSB) influences undernutrition and overnutrition among young children (14). Robertson's et al. paper “The Human Microbiome and Child Growth–First 1,000 Days and Beyond” found that an “undernourished” microbiome is intergenerational, thereby perpetuating growth impairments into successive generations, which may contribute to lifelong and intergenerational deficits in growth and development (15). The results of the above papers have greatly promoted the research on children's health, and have been cited by relevant scholars for many times. Highly cited papers (HCPs) in the Essential Science Indicators database refer to papers with citations in the top 1% of all papers in a research field, and they are considered to be symbols of scientific excellence and top performance of the past 10 years (16). The identification of HCPs on child nutrition could reflect the research progress and hot topics in this field accurately, which has an important reference to relevant scholars (16, 17). Bibliometric analysis is description of the external characteristics of the literature through mathematical and statistical methods, mainly based on the content of published journal papers as the main research object, and descriptive statistics on the academic status, such as journal distribution and main research institutions. Bibliometric analysis is one of the more effective research methods for evaluating the development stage of the discipline and predicting the future development trend (18, 19).

## Methodology

### Data Source and Search Strategy

We searched the Science Citation Index-Expanded (SCI-E) database on February 7, 2020. The specific retrieval strategy can be found in the Supplementary Material. The initial search yielded a total of 214,264 papers in the period from 1980 to the present. Among them, we chose the selection of “highly cited” in the field. Finally, 1,398 researches were included. There are no limitations on language, publication year, data category, and document type.

### Analysis Method

In this research, CiteSpace and VOSviewer tools were used to analyze the publication characteristics, including paper type, language and quantity, active authors, countries and institutions, journals, co-cited journals and co-cited references, co-occurrence keywords and burst keywords, and form social network maps (SNMs) based on the characteristics of the papers published (20–23). Due to the particularity of data format requirements of CiteSpace software, the selected literature was exported in the format of “RefWorks,” the data was saved in the format of “Download_XXX,” and imported into CiteSpace. Set the “Years Per Slice” length to “1,” the “Terms Types” to “Burst Terms,” and the “Pruning” to “Pathfinder.” Meanwhile, the selected literature was downloaded in the format of “TXT” and imported into VOSviewer software. The data type was set to “Create a map based on bibiographic data” and the data source was set to “Read data from bibliographic database files.” Different nodes represent different elements such as authors, countries, institutions, and keywords in a cluster map. The size of nodes indicates the number of publications or co-occurrence times of keywords. The lines between nodes reflects the relations of cooperation, co-occurrence, or co-citations. Nodes and lines of the same color represent the same cluster (24, 25). Microsoft Excel 2016 was used to conduct data aggregation and analysis.

## Results

### Paper Type, Language and Quantity

A total of 1,398 HCPs were retrieved from SCI-E, which includes 944 (67.525%) full-length research articles and 454 (32.475%) reviews. Most of the papers 1,394 (99.714%) were published in English, followed by 2 (0.143%) were published in German and 2 (0.143%) were published in Spanish.

**Figure 1:**
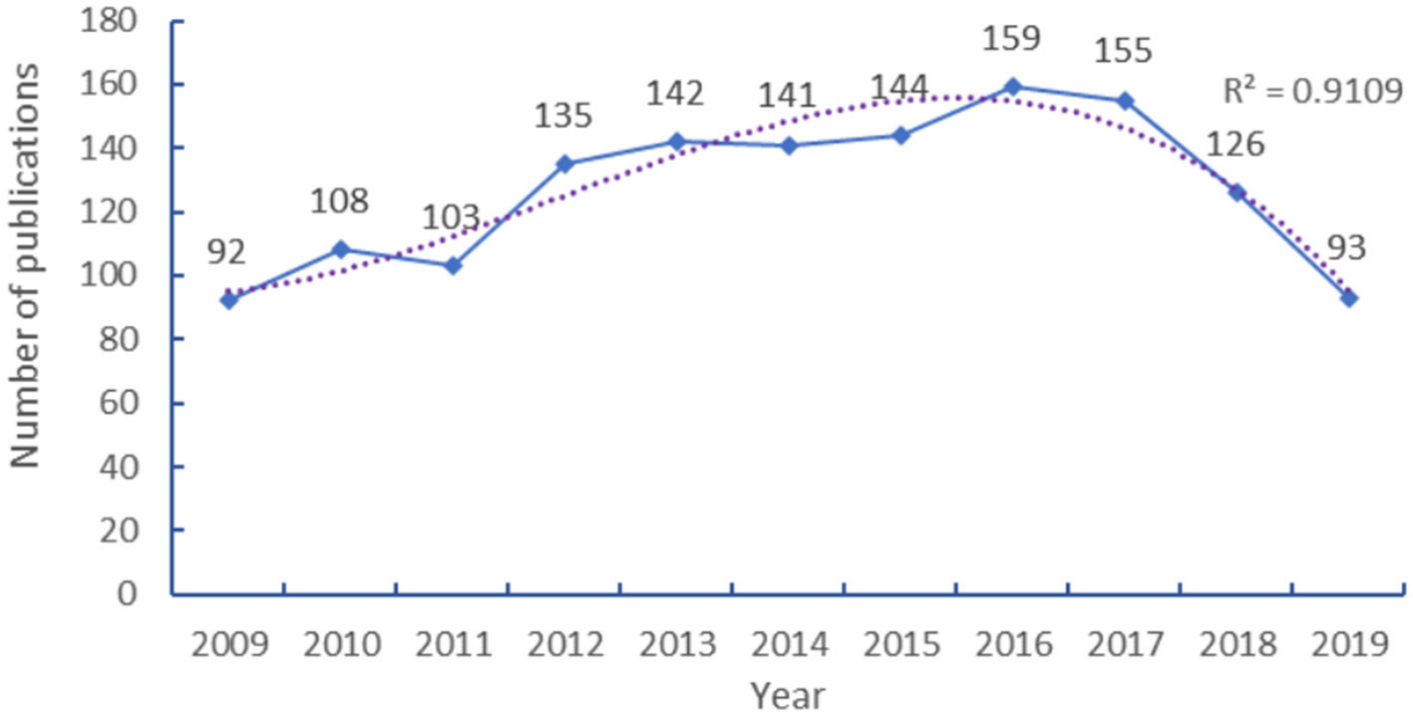
Publication years for HCPs on child nutrition.

The HCPs on child nutrition were published in 2009 (92 papers) and exceeded 100 papers in 2010 (Figure 1). In 2011, the number of publications decreased (103 papers). Since 2012, the number of publications increased by more than 30 (135 papers) and the growth rate continues slowly and steadily to 2017 (155 papers). The number of publications dropped significantly in 2018 and <100 papers in 2019 (Incomplete statistics). In this study, the relationship between the publication year and the number of publications is described using a polynomial model. There is a significant correlation between the number of studies and the year with a high coefficient of determination (*R*^2^ = 0.9109).

### Active Authors, Countries, and Institutions

The HCPs on child nutrition included 7,868 authors. The top 10 authors and co-cited authors were shown in Table 1. Tremblay has published the most, 17 (1.22%) papers, following Victora with 14 (1.00%) publications. The third were Lawlor and Bhutta both 12 publications (0.86%), following Black 11 publications (0.79%) and Smith 10 publications (0.72%). The other 4 authors all published fewer than 10 publications. The highest co-cited author was World Health Organization (621 co-citations), the remaining co-cited authors were Ogden (183 co-citations), Victora (135 co-citations), Deonis (132 co-citations), Cole (127 co-citations), Sallis (119 co-citations), Flegal (112 co-citations), and Kramer (107 co-citations). Other co-cited authors both 100 co-citations.

**Table 1:** The top 10 authors and co-cited authors [*n* (%)].

**Rank**	**Author**	***N* (%)**	**Country**	**Co-cited author**	**Country**	**Citations**
1	Tremblay, MS	17 (1.22)	Canada	World Health Organization	–	621
2	Victora, CG	14 (1.00)	Brazil	Ogden, CL	USA	183
3	Lawlor, DA	12 (0.86)	UK	Victora, CG	Brazil	135
4	Bhutta, ZA	12 (0.86)	Canada	de Onis, M	Switzerland	132
5	Black, RE	11 (0.79)	USA	Cole, TJ	UK	127
6	Smith, GD	10 (0.72)	UK	Sallis, JF	USA	119
7	Chaput, JP	9 (0.64)	Canada	Flegal, KM	USA	112
8	Daniels, SR	9 (0.64)	USA	Kramer, MS	Canada	107
9	Katzmarzyk, PT	8 (0.57)	USA	Black, RE	USA	100
10	Ogden, CL	8 (0.57)	USA	Holick, MF	USA	100

**Figure 2:**
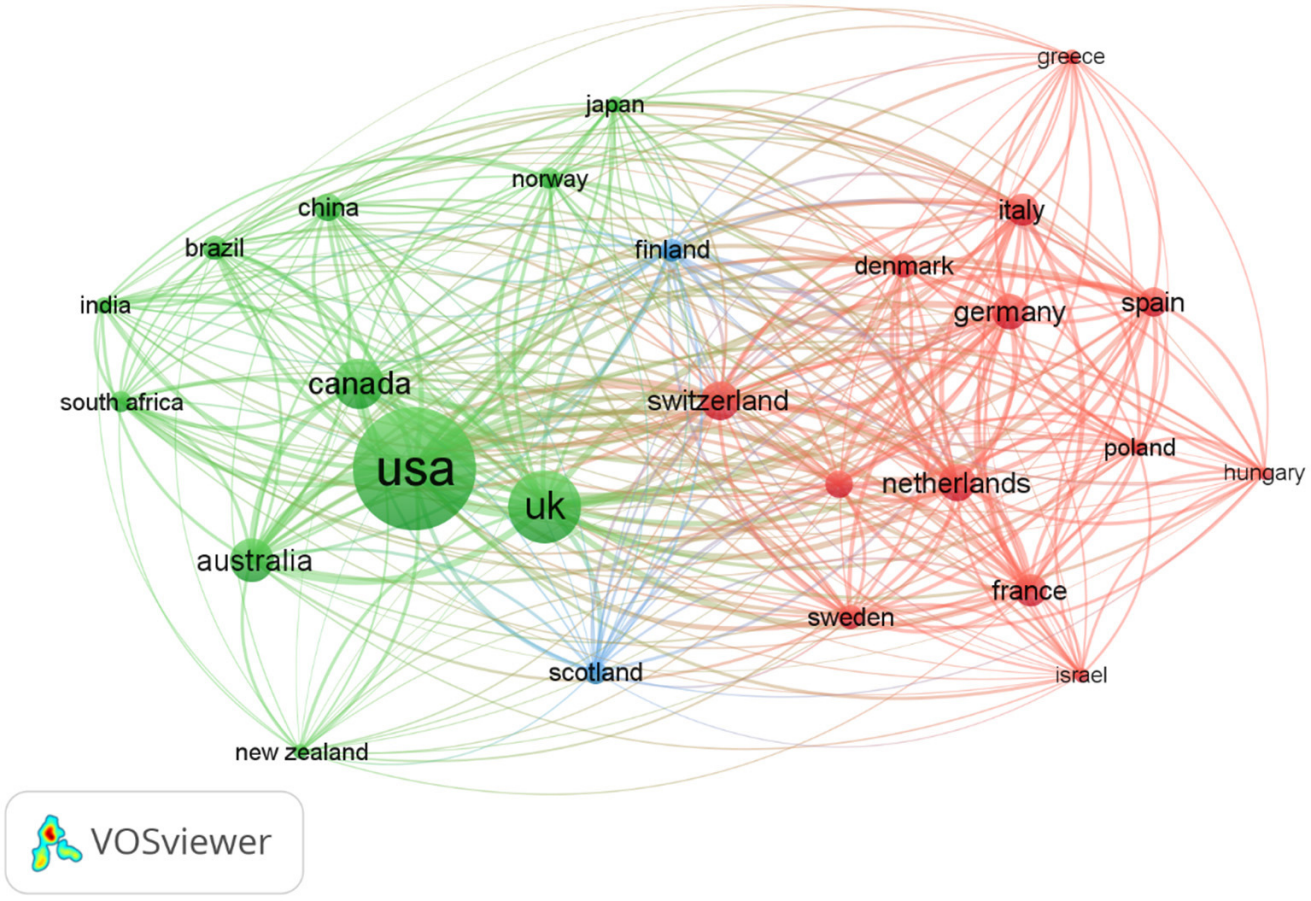
The network map of countries.

In total, 113 countries published papers in this study. The top 10 countries and institutions were shown in Table 2, with the USA ranked first, accounting for 59.87% and the UK ranked second (362 publications, 25.89%), followed by Canada (200 publications, 14.31%), Australia (161 publications, 11.52%), Switzerland (133 publications, 9.51%), Netherlands (127 publications, 9.08%), Germany (113 publications, 8.08%), France (101 publications, 7.22%). The other countries Italy and Spain both published <100 publications. As shown in Figure 2, the 59 countries with more than 4 papers were divided into 4 categories, with close cooperation among them.

**Table 2:** The top 10 countries and institutions [*n* (%)].

**Rank**	**Country**	***N* (%)**	**Institution**	***N* (%)**
1	USA	837 (59.87)	Harvard University	85 (6.08)
2	UK	362 (25.89)	University of North Carolina	56 (4.01)
3	Canada	200 (14.31)	University of Washington	48 (3.43)
4	Australia	161 (11.52)	Center for Disease Control and Prevention	43 (3.07)
5	Switzerland	133 (9.51)	University of Toronto	41 (2.93)
6	Netherlands	127 (9.08)	University of California, San Francisco	40 (2.86)
7	Germany	113 (8.08)	World Health Organization	40 (2.86)
8	France	101 (7.22)	Duke University	39 (2.79)
9	Italy	96 (6.87)	Emory University	39 (2.79)
10	Spain	83 (5.94)	University of Pennsylvania	38 (2.72)

Countries and institutions with greater influence and the status of cooperation could be displayed through the network maps. Two thousand four hundred eighty-two institutions contributed to the publications of this research. Table 3 showed the top 10 institutions. Harvard University ranked first (85 publications, 6.08%), followed by University of North Carolina (56 publications, 4.01%), University of Washington (48 publications, 3.43%), Center for Disease Control and Prevention (43 publications, 3.07%), University of Toronto (41 publications, 2.93%). The University of California, San Francisco and the World Health Organization published the same amount of papers (40 publications, 2.86%). Duke University and Emory University published the same amount of papers (39 publications, 2.79%). The 10th University of Pennsylvania published 38 (2.72%).

**Table 3:** The top 10 journals and co-cited journals [*n* (%)].

**Rank**	**Journals**	***N* (%)**	**Citations**	**Citation/*N*** **(average citation)**	**Country**	**IF (2019)**	**Co-cited journals**	**Co-citation**	**Country**	**IF (2019)**
1	Lancet	95 (6.80)	61,591	648.33	UK	60.392	Lancet	2,994	UK	60.392
2	New England Journal of Medicine	67 (4.79)	22,337	333.39	UK	74.699	American Journal of Clinical Nutrition	2,620	USA	6.766
3	Pediatrics	43 (3.08)	11,975	278.49	USA	5.359	Pediatrics	2,581	USA	5.359
4	Journal of American Medical Association	37 (2.65)	18,652	504.11	USA	45.540	New England Journal of Medicine	1,889	UK	74.699
5	Cochrane Database of Systematic Reviews	29 (2.07)	6,415	221.21	UK	7.890	Journal of Allergy and Clinical Immunology	1,575	USA	10.228
6	International Journal of Behavioral Nutrition and Physical Activity	27 (1.93)	5,510	204.07	UK	6.714	Journal of the American Medical Association	1,504	USA	45.540
7	Obesity Reviews	26 (1.86)	5,734	220.54	UK	7.310	International Journal of Obesity	1,247	UK	4.419
8	Journal of Allergy and Clinical Immunology	21 (1.50)	7,290	347.14	USA	10.228	PLoS One	1,178	USA	2.740
9	American Journal of Preventive Medicine	20 (1.43)	3,407	170.35	USA	4.420	Journal of Nutrition	1,089	USA	4.281
10	International Journal of Epidemiology	20 (1.43)	4,281	214.05	UK	7.707	Journal of Pediatric Surgery	1,025	USA	1.919

**Figure 3:**
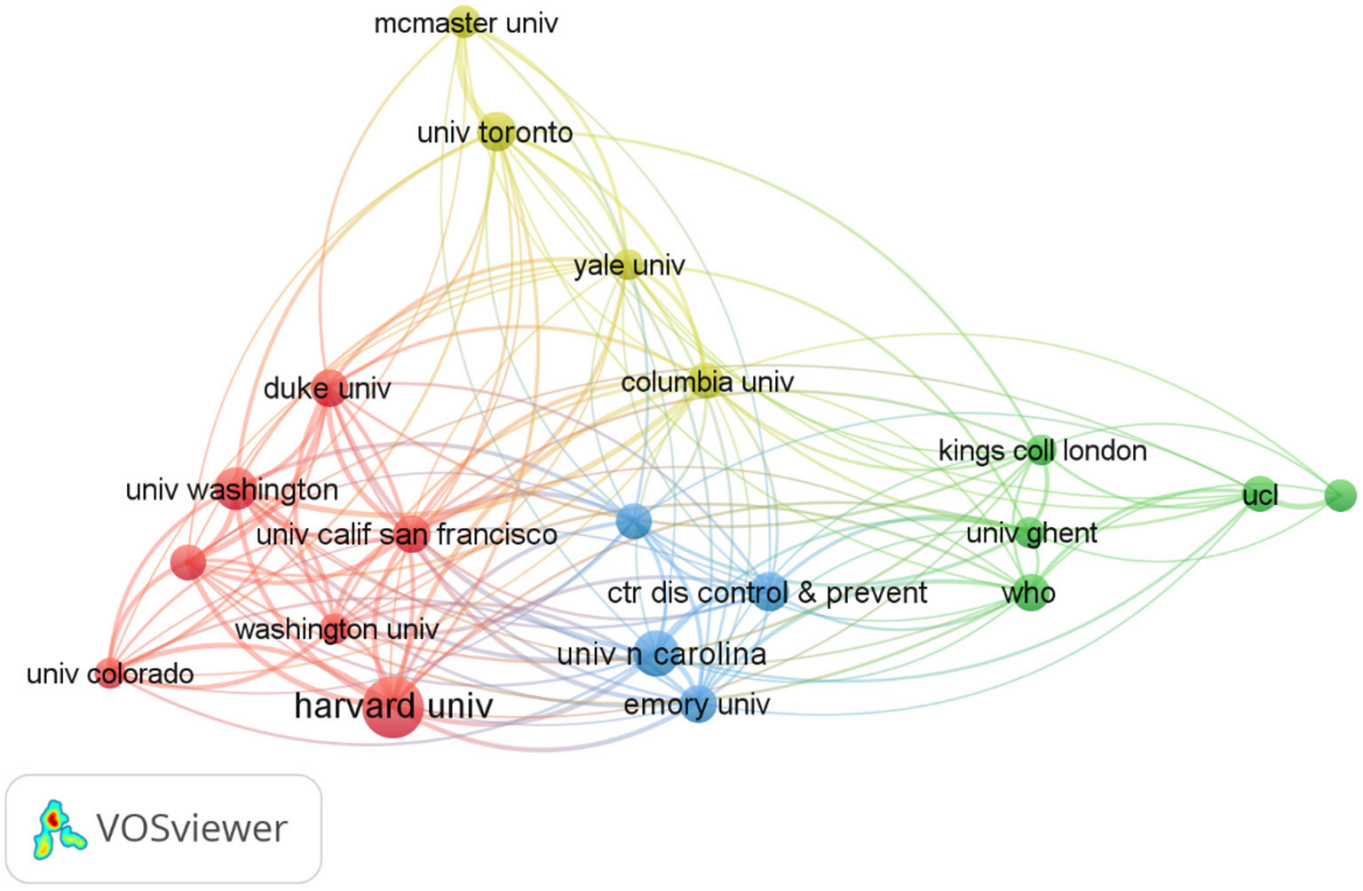
The network map of institutions.

The cooperation of institutions with more than 10 papers was shown in Figure 3. Major research institutions were divided into 5 clusters, and close collaboration between the groups.

### Journals, Co-cited Journals and Co-cited References

The study was published in 423 journals. The top 10 journals and co-cited journals, as well as the journal citation frequency, publishing countries and impact factor (IF) in 2019 could be seen in Table 3. The most published journal was *Lancet* (95 publications, 6.8%), following *New England Journal of Medicine* (67 publications, 4.79%), *Pediatrics* (43 publications, 3.08%), *Journal of American Medical Association* (37 publications, 2.65%), *Cochrane Database of Systematic Reviews* (29 publications, 2.07%). Six of the top 10 journals were from the UK and others from the USA. “Citation/N” represents the average citations per paper. The *Lancet* ranked the highest because the average citation frequency of papers published in the journal was 648.33 times.

**Figure 4:**
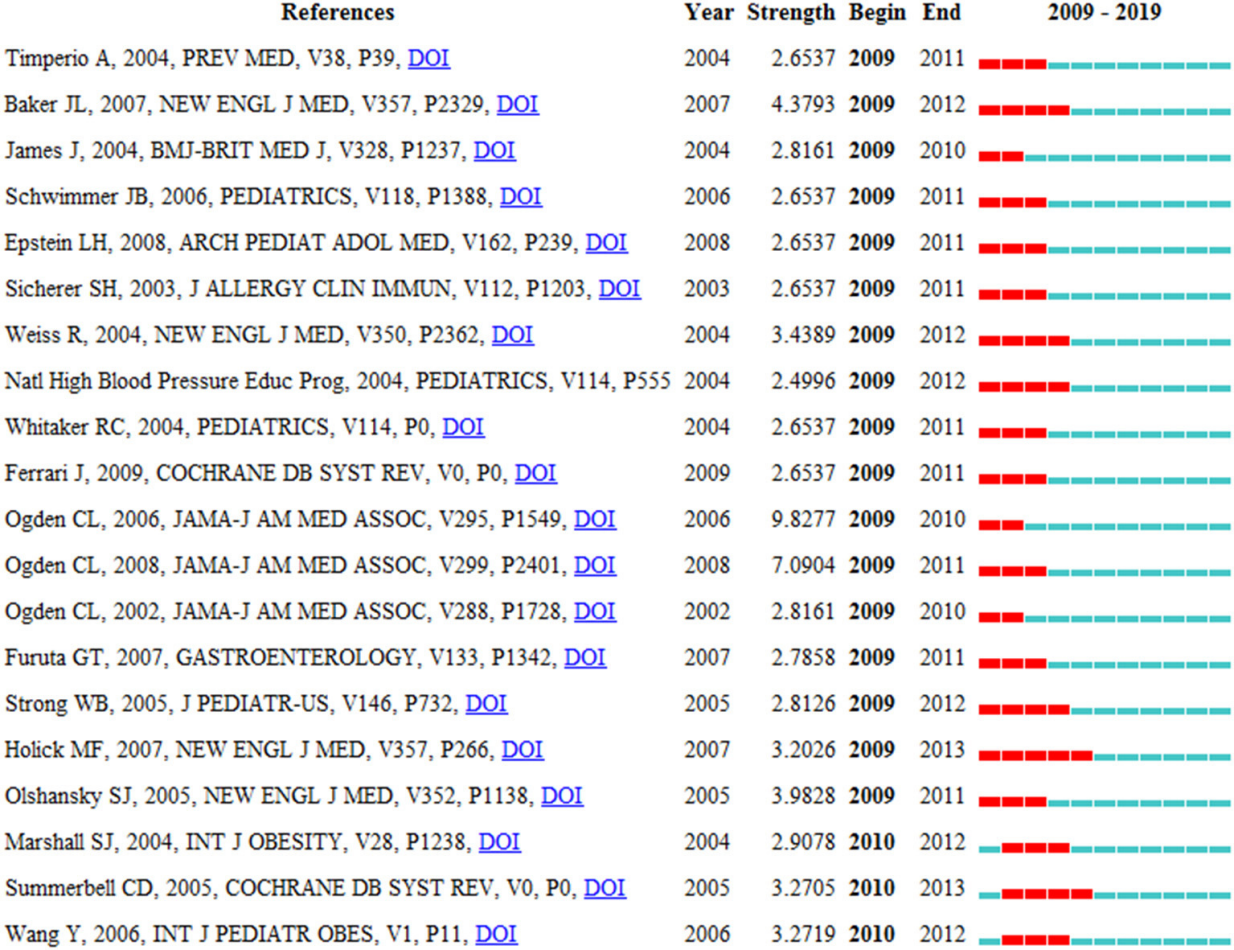
Top 20 references with the strongest citation burst.

*Lancet* was the most co-citation (2,994 co-citations), followed by *The American Journal of Clinical Nutrition* (2,620 co-citations) and *Pediatrics* (2,581 co-citations). Among the top 10 co-cited journals, 7 are from the USA. Table 4 shows the top 10 co-cited references related to this research. The co-citation can reflect the researchers' attention. One paper was co-cited more than 60 times (26). Two papers were co-cited between 35 and 60 times (27, 28). Others were co-cited between 30 and 35 times (29–35). The top 20 HCPs are shown in Figure 4. Papers with a high frequency of citations are represented by red bars, and with a low frequency of citations are represented by green bars. The first reference with citation bursts appeared in 2009, 85.00% were first discovered between 2009 and 2011. After 2009, 3 HCPs were detected with citation burst.

**Table 4:** Top 10 co-cited references.

**Rank**	**Co-cited reference**	**Co-citation**
1	Black, R. E. (2008). Maternal and child undernutrition: global and regional exposures and health consequences. *Lancet*. 371:243–60 (26)	63
2	Black, R. E. (2013). Maternal and child undernutrition and overweight in low-income and middle-income countries. *Lancet*. 382:427–51 (27)	38
3	Cole, T. J. (2000). Establishing a standard definition for child overweight and obesity worldwide: international survey. *BMJ*. 320:1240–63 (28)	37
4	Egger, M. (1997). Bias in meta-analysis detected by a simple, graphical test. *BMJ*. 315:629–34 (29)	34
5	Ng, M. (2014). Global, regional, and national prevalence of overweight in children and adults during 1980-2013: a systematic analysis for the Global Burden of Disease Study 2013. *Lancet*. 384:766–81 (30)	34
6	Ogden, C. L. (2006). Prevalence of overweight and obesity in the United States, 1999-2004. *JAMA*. 295:1549–55 (31)	33
7	Ogden, C. L. (2015). Prevalence of Childhood and Adults in the United States, 2011-2012. *JAMA*. 311:806–14 (32)	32
8	Turnbaugh, P. J. (2006). An obesity-associated gut microbiome with increased capacity for energy harvest. *Nature*. 444:1027–31 (33)	31
9	Victora, C. G. (2008). Maternal and child undernutrition: consequence for adult health and human capital. *Lancet*. 371:340–57 (34)	31
10	Yatsunenko, T. (2012). Human gut microbiome viewed across age and geography. *Nature*. 486:222–57 (35)	30

### Co-occurrence Keywords and Burst Keywords

**Figure 5:**
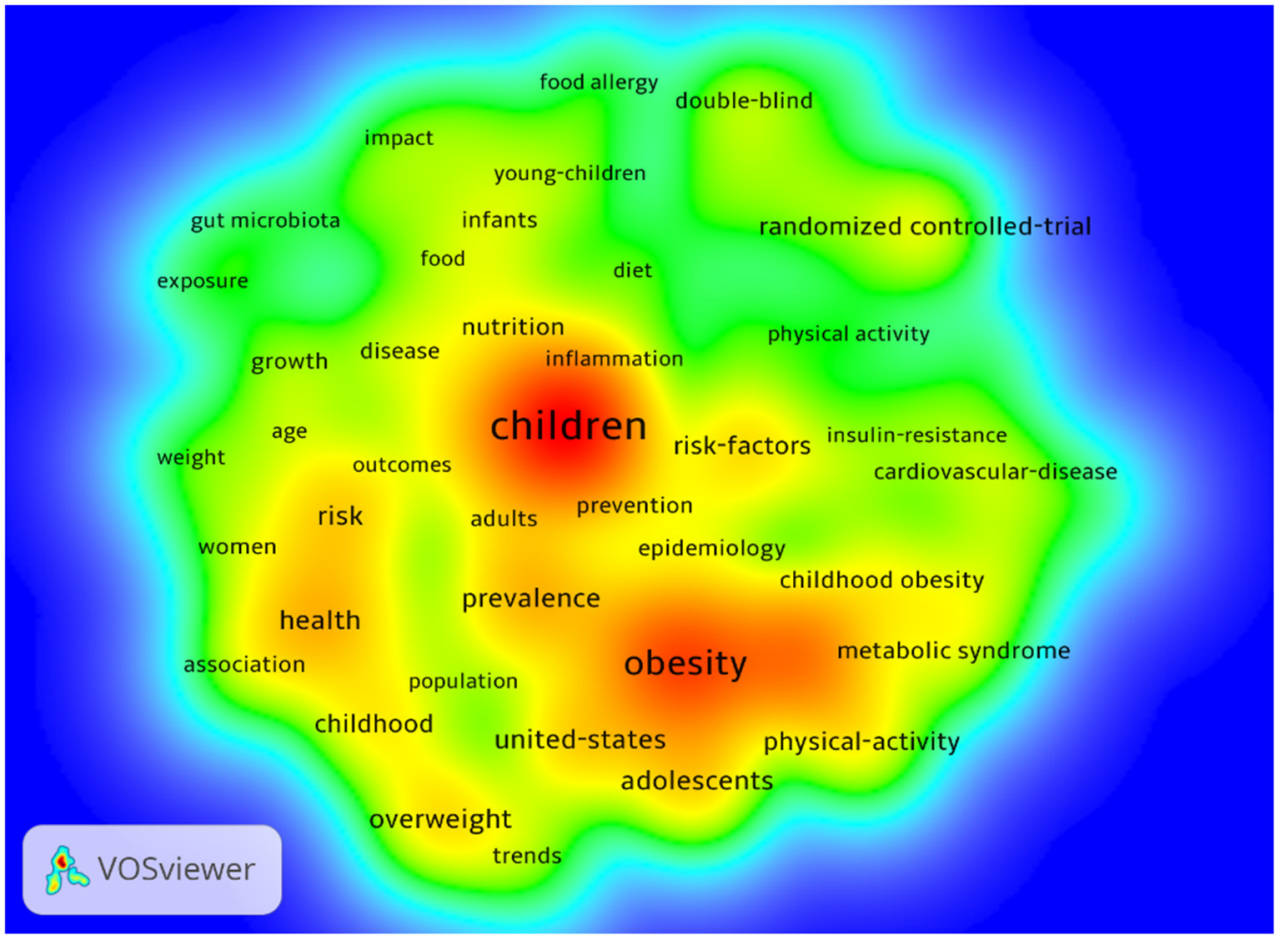
The density map of keywords.

We summarized and counted the keywords from the 1,398 HCPs. Figure 5 showed the visualization of color spectral density based on keywords and hotspot intensity, where warm red represents the hot areas and cold blue represents the cold areas. Children, obesity, health, prevalence, risk, risk-factors, metabolic syndrome, united-states, physical-activity, and adolescents were the keywords with the highest density.

**Figure 6:**
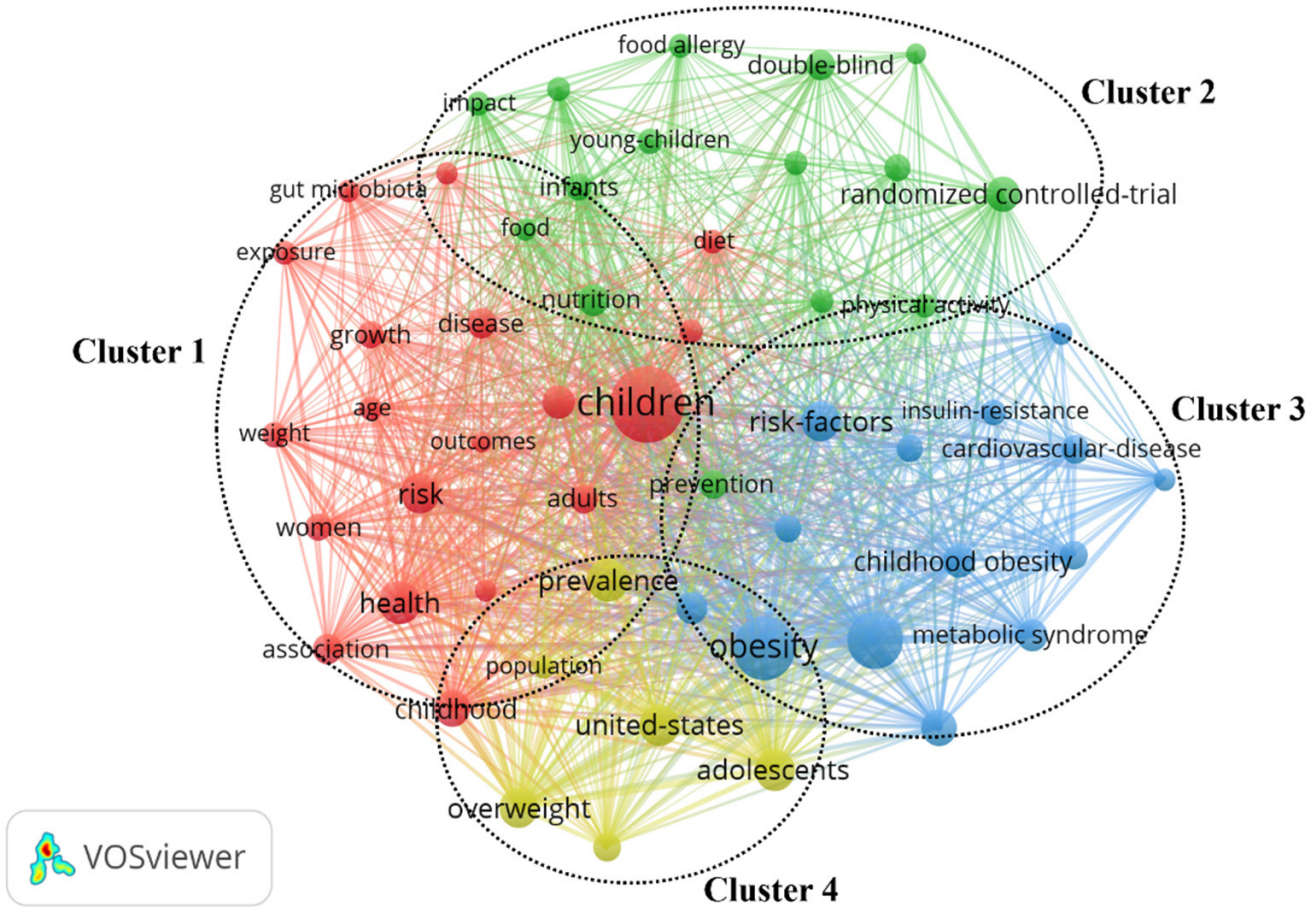
The network map of keywords.

The cluster map of the main keywords was shown in Figure 6. Four clusters were formed by these keywords. Cluster 1 was the largest of the four clusters, including 19 keywords, mainly focused on intestinal flora and physical health in children. Cluster 2 included 15 keywords, primarily focused on children's food intake and the prevention of food allergies. Cluster 3 included 14 keywords, mainly focused on the adverse outcomes of children who are overweight or obese. Cluster 4 included 6 keywords, mainly focused on the prevalence of overweight children in the USA.

**Figure 7:**
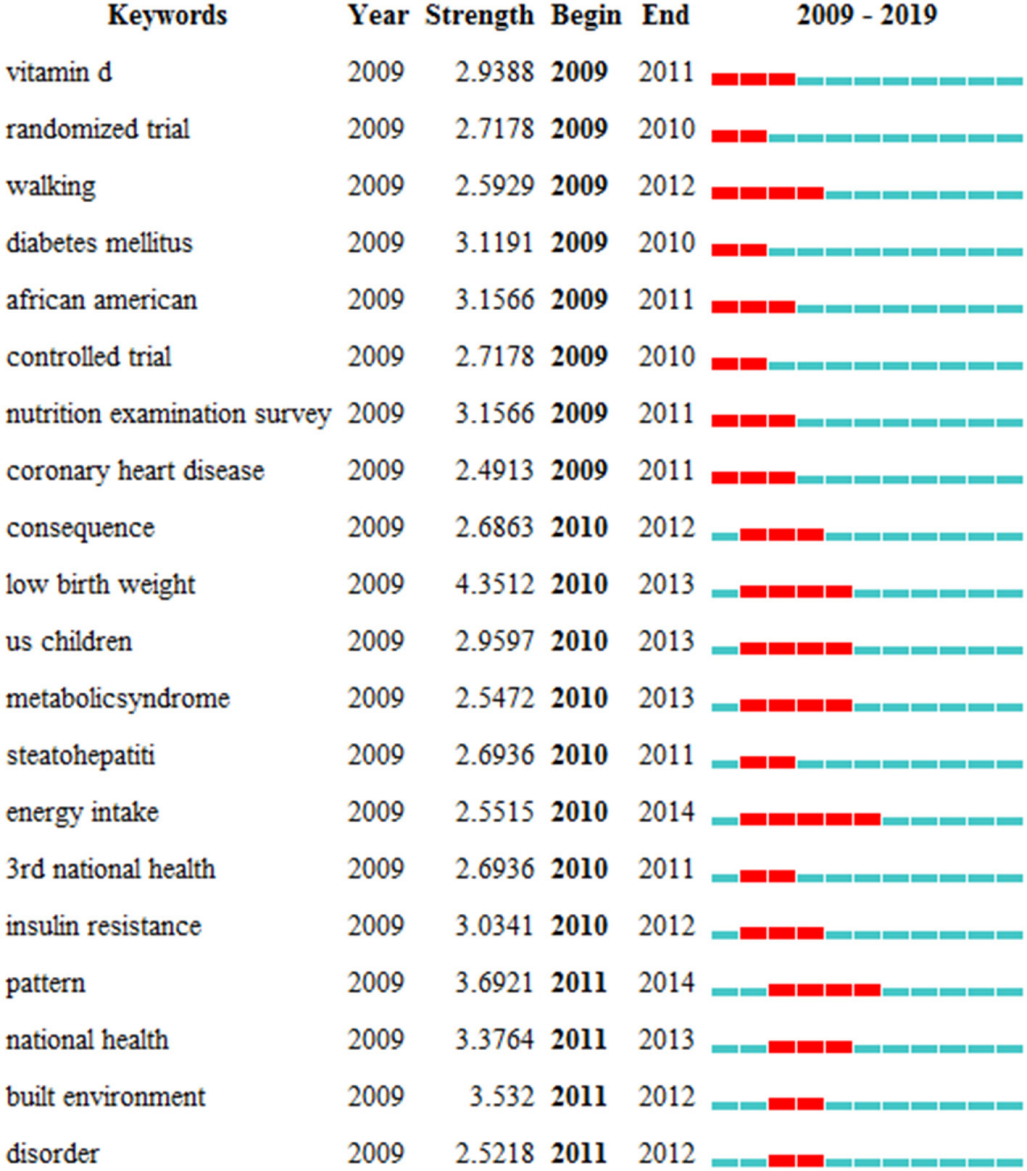
Top 20 keywords with the strongest citation burst.

The map of burst keywords was shown in Figure 7 to identify hot topics. The time period that represents the strongest citation bursts was indicated in red bar. Among them, 8 keywords were detected in 2009. In this period, Vitamin D supplements, obesity-related complications such as diabetes, coronary heart disease, and the National Health and Nutrition Examination Survey (NHANES) findings were hot research topics. From 2010 to 2014, metabolic syndrome, steatohepatitis, insulin resistance, low birth weight and the establishment of models and environments to promote children's health, were major concerns. After 2014, no prominent keywords were detected.

## Discussion

### Analysis of Paper Types and Publication Year

There were two types of HCPs included in this study, with reviews accounted for 32.475%. This phenomenon means that a lot of summaries and conclusions has been completed by researchers on the basis of existing research. This was undoubtedly great progress. Thus, we were confident that the number of studies on child nutrition will continue to increase, with greater content in the future. This trend will create more awareness and draw attention to children's nutrition and health globally.

The included HCPs were published from 2009 to 2019. Before 2012, the number of published HCPs was in a state of slow growth, and <130, which suggested that the development of child nutrition research was slow during the period, and researchers did not realize the importance of children's nutrition and health. After 2012, HCPs began to growth slowly and reached peak of 159 in 2016. This may be due to the fact that the WHO Child Growth Standards demonstrate that, by the time children reach the age of five, differences are more affected by nutrition, feeding methods, environment, and health care than by genetic or racial characteristics, and the Ninth Global Conference on health promotion focused on global childhood obesity (36, 37). After 2016, the number of HCPs has shown a decreasing trend. In general, the number of publications ranged from 90 to 160 annually.

### The Geographical Distribution of Research Group

Among 1,398 HCPs included, 7,868 authors involved. But only 6 (0.08%) authors have published more than 10 papers. Sixty-one (0.78%) authors have published more than 5 papers. Statistical results showed that 6,865 (87.25%) authors published only 1 paper. This reflects that few researchers have been committed to child nutrition and health.

Among the top 10 authors, 3 from Canada, Tremblay, Bhutta, and Chaput. They have published 38 HCPs in total. The reason might be that Tremblay, as a leader, has published many papers on the prevalence and long-term changes in overweight and obesity about Canadian children and adolescents. Tremblay and Chaput have been working closely together and have published several papers on children's physical activity to control overweight and obesity. Among them, *Systematic review of sedentary behavior and health indicators in school-aged children and youth*, published by Tremblay et al. was cited the most (38), more than 800 times. Among the top 10 co-cited authors, 5 from the USA, and the total number of citations was 614. *Prevalence of Childhood and Adult Obesity in the United States, 2011–2012*, published by Ogden et al. (32); *Prevalence and Trends in Obesity Among US Adults, 1999–2008*, published by Flegal et al. (39); *Maternal and child undernutrition and overweight in low-income and middle-income countries*, published by Black et al. (27); *Evaluation, Treatment, and Prevention of Vitamin D Deficiency: an Endocrine Society Clinical Practice Guideline*, published by Holick et al. (40) has been cited more than 1,000 times. This shows that the active and influential scholars are from Canada and the USA. Interestingly, neither the top 10 authors nor the co-cited authors have any scholars from China. Probably because: (1) Chinese scholars pay little attention to child nutrition; (2) The number and quality of papers published by Chinese scholars on child nutrition were small; (3) Chinese scholars had low English proficiency and obvious language barriers. Therefore, in the future, it is important for Chinese scholars to strengthen exchanges and cooperation with outstanding foreign scholars to learn advanced research methods, broaden their horizons and ideas.

Although child nutrition deserves global attention, 24.11% of countries have only 1 highly cited paper. Among the top 10 countries with HCP on child nutrition, all included developed countries and no developing countries, which indicates that developing countries are lagging in this field. Among the top 10 institutions, 8 from the USA and 1 from Canada. This phenomenon showed that the USA was in a dominant position and there was a large gap between developing and developed countries in child nutrition research. In short, the global impact of developing countries on child nutrition is limited. There was need for collaborative efforts between high income countries and Low and Middle income countries (LMIC) to improve and carry out more high impact research in the areas of child nutrition especially among children in LMIC.

In terms of cooperation between countries, both the USA and the UK maintain close cooperation with 55 countries, Canada and Switzerland with 54 countries, respectively, followed by Australia and Belgium with 53 and 50 countries, respectively, while Slovakia did not cooperate with any country. It was noteworthy that, China has partnerships with 48 countries, so we are confident that China will be at the forefront on child nutrition in the future.

### Analysis of Published Sources

The included 1,398 HCPs were published in 423 journals, each journal should publish an average of 3.30 papers, but in fact, 6.38% of journals published more than 10 papers and 58.16% of journals only published 1 paper. Four major journals published 242 papers, accounting for 17.32%. These journals were as follows: *Lancet* (6.80%), *New England Journal of Medicine* (4.79%), *Pediatrics* (3.08%), *Journal of American Medical Association* (2.65%). The *Lancet* was the journal with the most productive and cited researches of HCPs on child nutrition. Among the authors, Black et al. (27) and Ng et al. (30) from the USA published many papers, and have been cited 2,568 times and 5,253 times, respectively. The unprecedented World Summit for Children, held at the United Nations headquarters in New York City in 1990, set out 10-year goals for children's health, nutrition and education (41); the special session on children held in 2002 reviewed the progress made in children's affairs since the 1990 World Summit for Children and reinvigorated the global commitment to children's rights (42). Through holding these international conferences, scholars have developed great interest on child nutrition, which has also aroused scholars' attention.

Among the top 10 journals, six were from the UK and none from China, which once again confirms the huge gap in scientific research between developing and developed countries. Citation/N is an important index to measure the scientific importance or quality of a paper. It also shows that the quality of the journal is high and the content is attractive. The American *Journal of Allergy and Clinical Immunology* performed well. This journal were recognized and welcomed by scholars, because although the journal published only 21 HCPs, the citation /N was high, which also shows the high academic influence of the journal.

### Analysis of the Main Keywords

#### Intestinal Flora and Physical Health

In this study, one of the important research hotspots and directions was the composition and roles of intestinal microbiota in children (Figure 6, cluster 1). There are a large number of bacteria in the human gut, which together make up the intestinal flora. In the human body, cells and bacterial cells are symbiotic, and there are 10 times as many bacterial cells as there are human cells (43, 44). The effects of intestinal flora on the human body are mainly manifested as nutrient absorption, substance metabolism, and immune defense (45). Some minerals, such as calcium, iron, and magnesium, are absorbed by the body through the intestinal flora (46). The intestinal flora is also involved in the metabolism of certain substances by fermenting food, synthesizing exercise fatty acids and vitamin K, which are then absorbed by the body (47). For children, intestinal bacteria can not only regulate the activity of cytotoxic T cells and natural killer cells, reduce the replication of viruses in cells, but also play an important role in innate immunity, activation of the immune system and the formation of an adaptive immune response (48, 49). Probiotics can significantly increase the expression of CD3 + CD4 + in children with severe HFMD caused by EV71, enhance the immune function of T cells and improve the cellular immune response of children. Therefore, intestinal flora plays an important role in children's growth. Gao et al. considered that the diversity of intestinal microflora in obese children was lower than that in normal children, and the relative abundance of intestinal flora at different classification levels was significantly different (50). Some studies have shown that the mode of delivery affects the bacterial community in the newborn gut. Guarino et al. noted in cesarean delivery, direct contact of the mouth of the newborn with vaginal and intestinal microbiota is replaced by exogenous non-maternally derived bacteria colonizing the infants' intestine producing a less diverse flora (51). Biasucci et al. believed that intestinal bacterial colonization of infants born by cesarean section is more likely to change (52). However, Rutayisire et al. considered that the diversity and colonization pattern of intestinal flora were significantly correlated with the mode of delivery 3 months before birth, but the difference disappeared after 6 months (53). Therefore, the diversity and colonization level of intestinal microflora and the mode of delivery as well as its extensive impact on the health of infants at all stages of life should be further studied.

#### Prevention of Food Allergies

Food allergies are common and affect about 8% of children in the United States. It brings a huge physiological, economic and social burden to children and families. There is no cure for food allergies (54). Therefore, the prevention and treatment of food allergy in children is also a key topic in recent years (Figure 6, cluster 2). Food allergies can be a variety of symptoms in children, with skin and gastrointestinal symptoms being the most common (55). Children under the age of 6 are often allergic to high-protein foods such as eggs, milk, peanut, and soy, as children's immune systems are not yet mature and the protective function of the gastrointestinal mucosa in infants is not perfect (56). Food allergies have a significant impact on the morbidity, living quality of infants and young children, which has become a concern for many parents (57). Peanut is one of the most common food allergies in children, which is becoming more and more common over time. So far, there is no effective treatment for peanut allergy, only through the use of epinephrine to avoid and alleviate this symptom. The double allergen exposure hypothesis suggested that the dermal sensitization of peanut may be the pathophysiological mechanism of peanut allergy development. In the future, oral and epicutaneous immunotherapy may be used as exciting tools to achieve peanut desensitization in children. In the past, people focused on the treatment of food allergy, but seldom considered the mental health consequences of living with the condition (58, 59). Feng et al. found that patients with food allergy may have depression, anxiety, post-traumatic stress, being bullied, and poor overall quality of life. At the same time, the patient's family life will also be disturbed (60). Parents of children with food allergies, especially mothers, report anxiety, depression, and decreased quality of life (61). Fong et al. stressed that children and adolescents with food allergies in the Australian population are vulnerable to bullying. It's a significant social problem that requires addressing to positively assist these children (62).

At present, in the treatment and prevention of food allergies, bacterial therapy has attracted more and more attention from scholars. They believe that one of the effective ways to prevent allergic diseases is fecal flora transplantation (63, 64).

#### Overweight or Obesity in Children

At present, childhood obesity is also widely concerned (Figure 6, cluster 3 and cluster 4). In 2017, the WHO announced that the number of obese children and adolescents aged 5 to 19 worldwide has increased ten-fold in the past 40 years. If the current trend continues, the number of obese children and adolescents will exceed the number of moderately or severely underweight by 2022 (64). American children's obesity rate ranks first in the world. Children with obesity may develop many serious comorbidities. These diseases include musculoskeletal diseases, cardiovascular diseases such as hypertension, insulin resistance and hyperlipidemia, respiratory diseases such as sleep apnea or asthma, and digestive system diseases such as non-alcoholic fatty liver disease (65, 66). Childhood obesity has a greater risk of persistence in adulthood. Low socioeconomic status, immigration background, and clinical susceptibility to obesity are the serious risk factors for obesity. However, the individual causes of obesity are quite complex, so it is necessary to make a systematic analysis of individual differences, and to formulate differentiated and realistic treatment plans. In addition to the rare monogenic or syndromic obesity, the treatment of childhood obesity should rely on professional lifestyle intervention programs. In general, a key component of a treatment strategy should include improving nutrition, physical exercise and self-esteem, while reducing stress. Besides, the inclusion of parents in treatment strategies has proved beneficial and necessary. Studies have shown that male children are 1.6 times more likely to be overweight/obesity than female children; children of overweight mothers are 3.34 times more likely to be overweight/obesity than children of normal weight mothers; preschool children's overweight/obesity is related to physical activity, screen time, eating snacks when watching TV, using computers, tablets and mobile phones (64).

Considering the individual differences between obese children and the complexity of obesity, there is no effective treatment for all groups. The most appropriate intervention method is determined by the age of children and the degree of overweight. The current methods of weight loss include lifestyle change interventions, bariatric surgery and drug use. Lifestyle change is the most widely used way to treat childhood obesity (67). This approach is designed to improve the quality of diet, increase physical activity and reduce sedentary behavior, usually using behavior change techniques to help maintain positive change and prevent recurrence. Many interventions focus on families, and parents are defined as “agents of change,” especially among children under 12 (68). Bariatric surgery generally includes gastric shunt, sleeve gastrectomy and gastric banding (69). Currently, the drugs used to treat obesity include: (1) Sibutramine, an appetite inhibitor, which is still allowed in Brazil, was suspended by the European drug agency in 2010 due to its adverse cardiovascular effects, and was withdrawn by the US Food and Drug Administration (FDA) in 2010; (2) Orlistat, a fat absorption inhibitor, has been approved by the FDA, but only for children under the age of 12. Other drugs often used to treat childhood obesity include the antidiabetic drug metformin and the antidepressant fluoxetine (67). New drugs for appetite regulation are currently under development or evaluation.

## Conclusions

At present, more and more researches on child nutrition have been published. The bibliometric method could be used to systematically analyze the characteristics of the papers and show the research status, hot spots, and future development trends. The results showed that 6,865 authors (87.65%) only published 1 paper. Scholars from the UK, the USA, and Canada had a greater academic influence. Scientific research institutions from the USA contributed the most. Strengthening academic exchanges and cooperation is the top priority for future development. Although great progress has been made, further research is needed to understand most of the unknown problems. Combining the above research results, the future development direction of children's nutrition research is put forward:

It has been concluded that the mode of delivery will affect the bacterial community in the intestinal tract of newborns. However, it is still controversial whether the adverse effects will last into childhood or even adulthood. Further research should be carried out in the future, because the results may affect pregnant women's choice of delivery mode.

Food allergies have a significant impact on the morbidity, living quality of children, but there is no effective treatment. The future research should focus on the induction of food allergy in children, the causes of sensitization, clinical manifestations, prognosis, precautions and so on, to solve this scientific problem.

Strengthen the research on the causes, types, prevention and targeted intervention measures of overweight or obesity in children. A large number of studies predict that the number of overweight or obese children will continue to increase in the future. Therefore, we should better understand the source of obesity and control the number and type of obesity children from the source.

## Strengths and Limitations

According to our knowledge, this study is the first bibliometric analysis of highly cited articles on child nutrition. Therefore, the study is original. This study not only provide a historical perspective for future research, but also highlight research areas requiring further investigation and development. In addition, before literature search, we read a large number of high-level papers and extracted search terms related to child nutrition. After integration, we formulated the search strategy for this study. Therefore, the search strategy is complete and scientific. Of course, this study also has some shortcomings, the WOS database is considered the most critical data source in bibliometric analysis, so we only searched it (70), some studies may have been overlooked. Besides, there are many authors in this study, some authors may have the possibility of renaming or having the same author from different institutions. Although we have carefully proofread the process, some mistakes are inevitable.

## Author Contributions

JW designed this study. YW, QL, and YC performed search and collected data and wrote the manuscript. YQ rechecked data. BP and QW performed analysis. LG rechecked. JW and GD reviewed the manuscript. All authors contributed to the article and approved the submitted version.

## Funding

This work was supported by the National Research Project Development Plan of Gansu Provincial Hospital (19SYPYB-18); Lanzhou Chengguan District Science and Technology Plan Project (2019RCCX0011).

## Conflict of Interest

The authors declare that the research was conducted in the absence of any commercial or financial relationships that could be construed as a potential conflict of interest.

## Publisher's Note

All claims expressed in this article are solely those of the authors and do not necessarily represent those of their affiliated organizations, or those of the publisher, the editors and the reviewers. Any product that may be evaluated in this article, or claim that may be made by its manufacturer, is not guaranteed or endorsed by the publisher.

## Abbreviations

HCPs: highly cited papers.
