# Supplementary material for: Global Trends and Future Prospects of Child Nutrition: A Bibliometric Analysis of Highly Cited Papers
Source: Front Pediatr. 2021 Sep 9;9:633525. doi: 10.3389/fped.2021.633525 (PMC8459025; doi:10.3389/fped.2021.633525)
Supplement: Supplementary file 1 [file Data_Sheet_1.docx]

**Supplementary Materials: Search strategy**

Database: *Web of Science* < *February 7th 2020>* 1398

Science Citation Index Expanded (SCI-EXPANDED) – from 1980 to now

| **#** | **Searches** | **Results** |
| --- | --- | --- |
| #1 | TS=(Nutritional Sciences OR Nutritional Physiological Phenomena OR Nutrition Assessment OR Nutrition Therapy OR Nutrition Policy OR Nutritional and Metabolic Diseases OR nutrition* OR diet OR feeding OR dietary OR breastfeed* OR breast feed* OR bottle feed* OR complementary feeding OR weaning OR Feeding Methods OR nutritional status OR overweight OR obese OR obesity OR overnutrition OR over nutrition OR undernourished OR overnourished OR underweight OR undernutrition OR under nutrition OR body weight OR food OR food and beverages OR vegetable* OR fruit* OR meat OR dairy OR dietary fat* OR starch* OR cereal OR micronutrient* OR vitamin* OR macronutrient* OR carbohydrate* OR dietary protein* OR saturated fat* OR unsaturated fat* OR mono unsaturated fat* OR monounsaturated fat* OR poly unsaturated fat* OR polyunsaturated fat* OR trans fat* OR dietary fibre OR dietary fiber OR dietary salt OR table salt OR soft drink OR fruit juice OR vegetable juice OR milk OR tea OR coffee OR energy drink* OR carbonated beverage* OR carbonated drink* OR calories OR caloric intake OR energy intake) | 3298305 |
| #2 | TI=("lactation" OR "enteral" OR "parenteral" OR "wasted" OR "wasting" OR "stunting" OR "stunted" OR "anthropometry" OR "Body Weights and Measures" OR "growth monitoring" OR "food labelling" OR "food assistance" OR "supplementary feeding" OR "diet therapy" OR "food-drug interactions" OR "food supply" OR feeding behavio* OR eating behavio* OR food pattern* OR "food hypersensitivity" OR "food deprivation" OR "food organic" OR "thiamin" OR "riboflavin" OR "niacin" OR "pantothenic" acid OR "pyridoxine" OR "pyridoxal" OR "pyridoxamine" OR "biotin" OR "folic acid" OR "folate" OR "cyanocobalamin" OR "choline" OR "retinol" OR "ascorbic acid" OR "tocopherol" OR "carotenoids" OR "carotene" OR "cryptoxanthin" OR "lutein" OR "lycopene" OR "zeaxanthin" OR "minerals" OR "calcium" OR "chloride" OR "magnesium" OR "phosphorus" OR "potassium" OR "sodium" OR "iron" OR "sulphur" OR trace element* OR "boron" OR "cobalt" OR "chromium" OR "copper" OR "fluoride" OR "iodine" OR "iron" OR "manganese" OR "molybdenum" OR "selenium" OR "zinc" OR trace metal* OR "prebiotics" OR "probiotics" OR "glycemic load" OR "glycemic index" OR "glycaemic load" OR "glycaemic index" OR "prebiotics" OR "probiotics" OR "glycemic load" OR "glycemic index" OR "glycaemic load" OR "glycaemic index" OR "kilocalories" OR "kilojoules") | 1436400 |
| #3 | #1 OR #2 | 4535650 |
| #4 | TI=child* | 700090 |
| #5 | #3 AND #4 | 214264 |
| #6 | #5 Select “Highly cited papers” | 1398 |
